# Supplementary material for: Prediction Power on Cardiovascular Disease of Neuroimmune Guidance Cues Expression by Peripheral Blood Monocytes Determined by Machine-Learning Methods
Source: Int J Mol Sci. 2020 Sep 2;21(17):6364. doi: 10.3390/ijms21176364 (PMC7503551; doi:10.3390/ijms21176364)
Supplement: Supplementary file 1 [file ijms-21-06364-s001.pdf]

Supplementary Materials

Supplemental table 1. NGC expression (mean and S.D.) of in patients and healthy subjects.

NGC	CVD Mean	CVD S.D.	Healthy Mean	Healthy S.D.
SEMA4D	11.70	0.23	11.80	0.16
PLXNB2	11.51	0.42	11.51	0.15
SEMA4A	11.34	0.49	11.41	0.21
SEMA4B	9.77	0.28	9.97	0.20
SEMA3E	9.25	1.09	9.98	1.11
NEO1	8.54	0.17	8.51	0.08
PLXNC1	8.32	0.50	7.81	0.22
ADORA2B	7.72	0.34	7.80	0.27
EPHB6	7.70	0.27	7.66	0.14
PLXND1	7.46	0.34	7.31	0.16
EFNA4	7.24	0.19	7.09	0.23
EFNB1	7.20	0.26	7.43	0.24
EPHB2	7.04	0.34	6.93	0.26
SEMA3C	6.85	0.27	6.92	0.29
EPHB4	6.83	0.31	6.65	0.31
SEMA6B	6.82	0.29	6.73	0.26
ROBO3	6.73	0.28	6.83	0.20
NRP1	6.56	0.18	6.50	0.13
NTNG2	6.54	0.28	6.46	0.30
DSCAM	6.41	0.19	6.19	0.16
SLIT2	6.34	0.15	6.22	0.11
PLXNA2	6.32	0.14	6.20	0.13
PLXNA1	6.29	0.26	6.23	0.24
PLXNA3	6.28	0.28	6.39	0.30
EPHB3	6.23	0.17	6.16	0.16
DCC	6.10	0.18	6.23	0.16
NRP2	6.08	0.14	6.02	0.11
EPHA2	6.01	0.12	6.09	0.12
EPHB1	5.99	0.14	5.94	0.12
EPHA3	5.98	0.11	6.04	0.10
SEMA6D	5.93	0.11	6.00	0.10
NTNG1	5.91	0.11	5.96	0.13
EFNA2	5.88	0.12	5.95	0.11
EFNB2	5.76	0.11	5.70	0.06
EPHA5	5.74	0.13	5.65	0.11

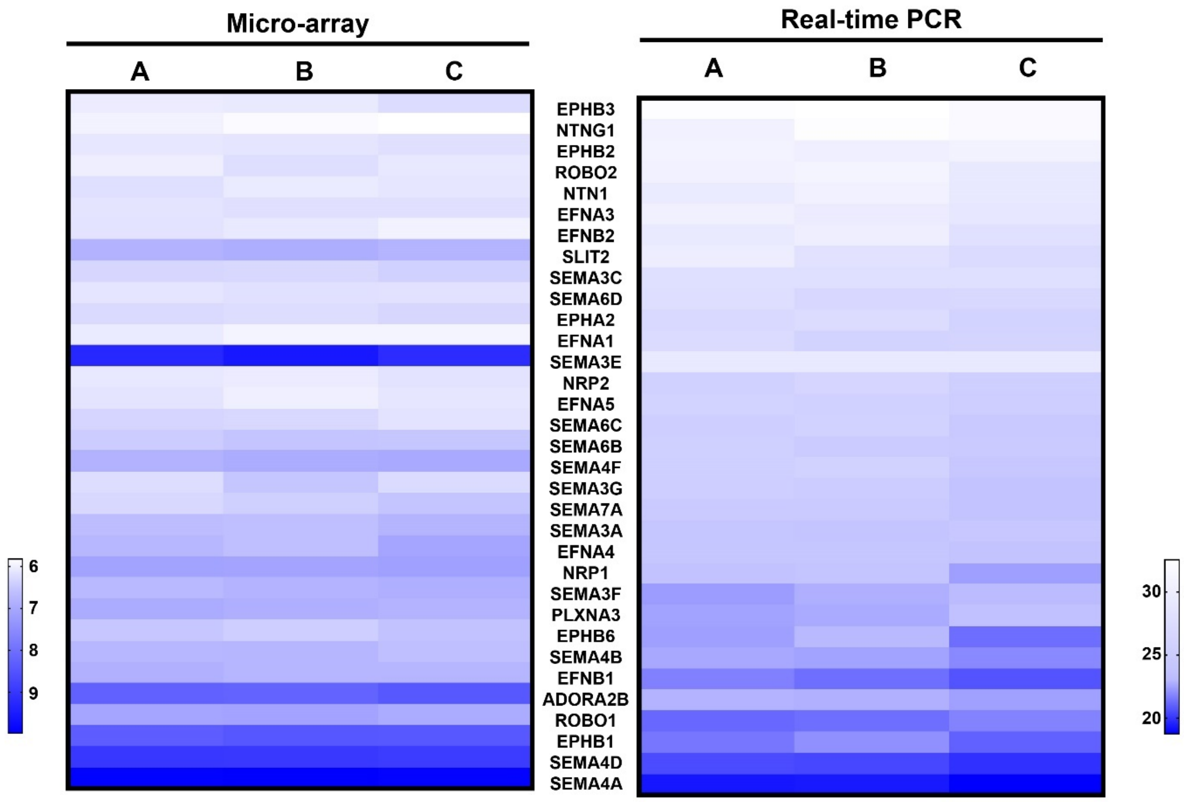

Supplemental figure 1. Comparison micro-array and real-time PCR. Heat-map of mRNA expression profiling of THP-1 cells for NGCs using micro-array and real-time PCR (n=3).

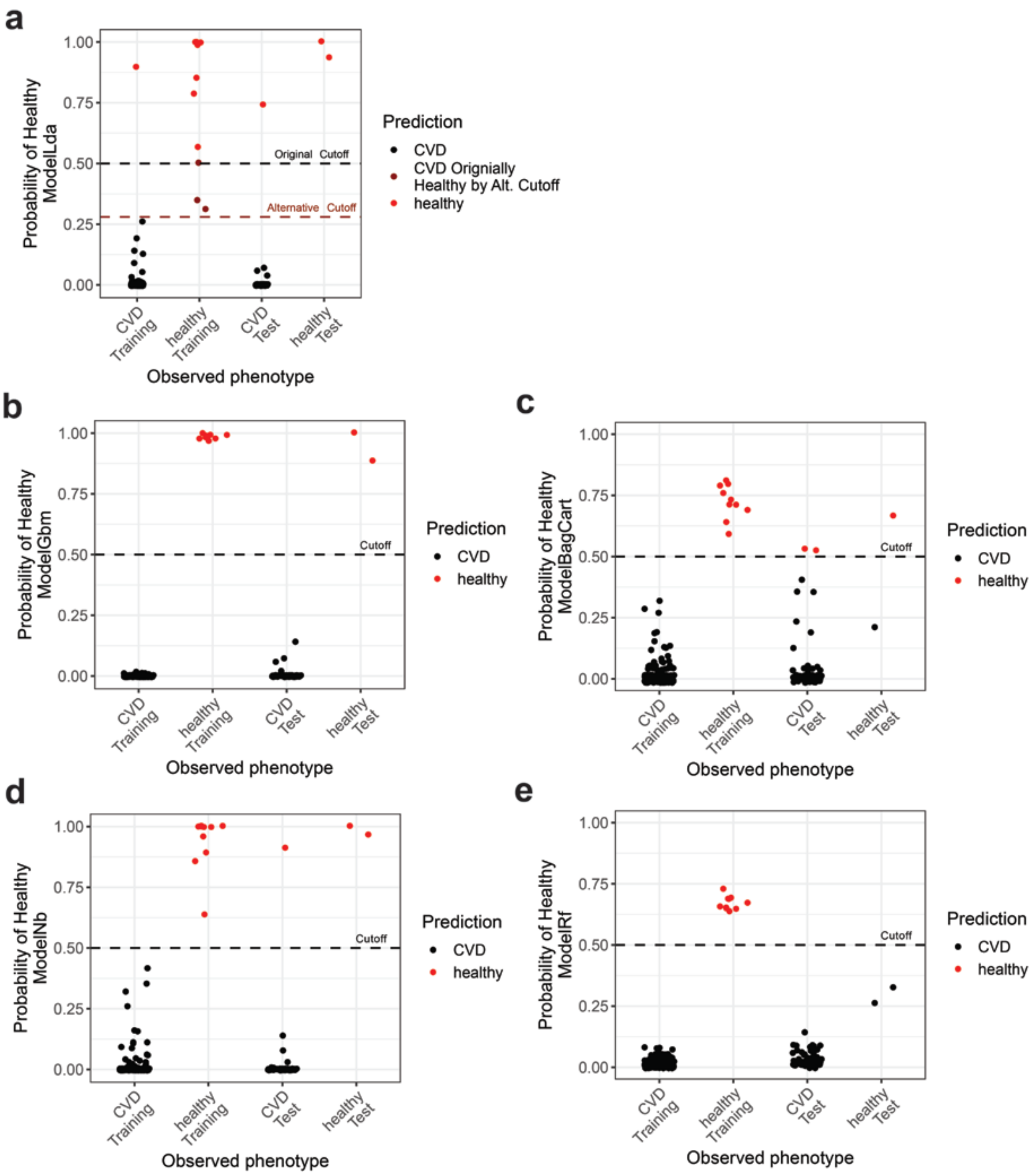

Supplemental figure 2. Probability distribution of Random Forrest and Bagged CART model.

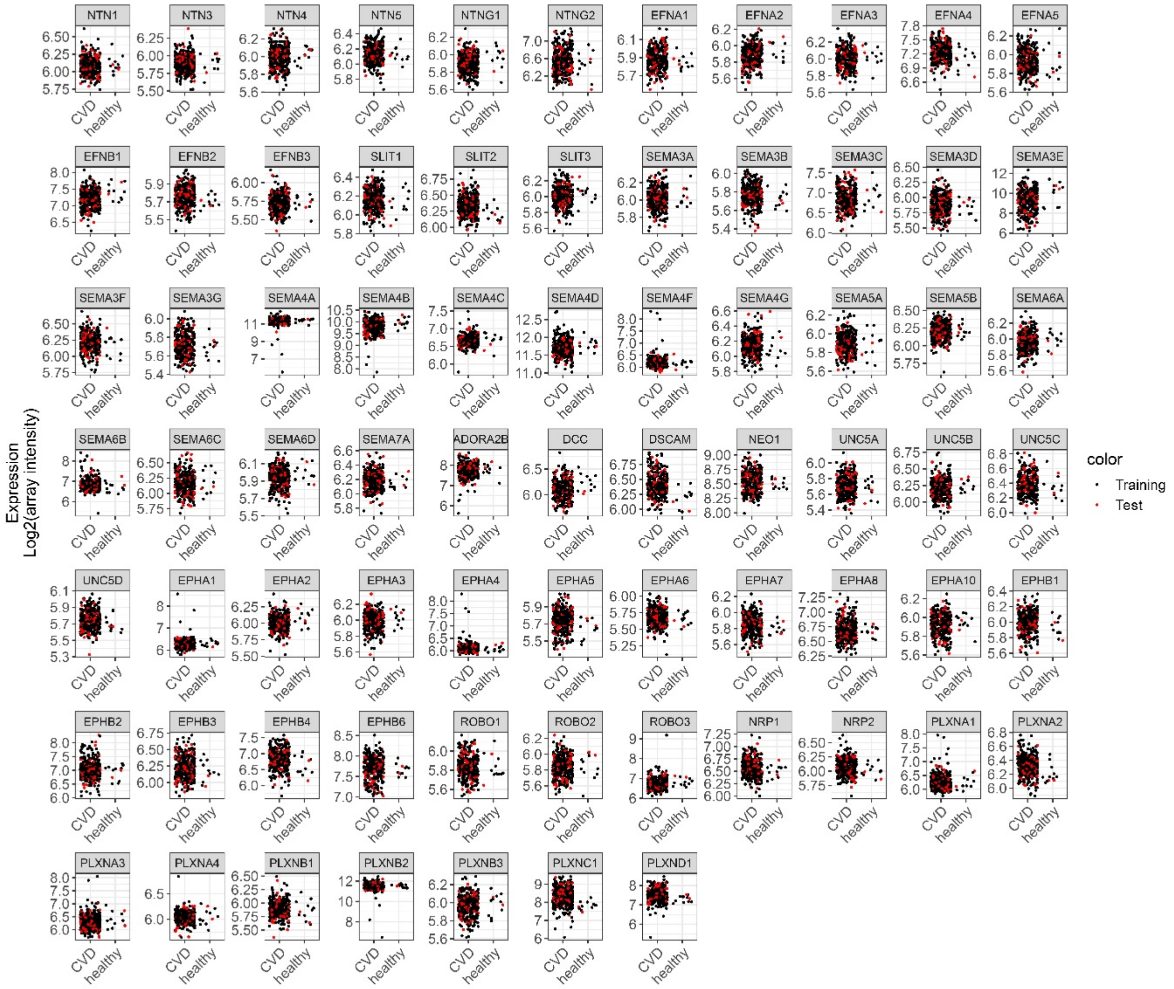

Supplemental figure 3. Distributions of NGC expressions in training set and test set. Jittered point plot of NGC expression in healthy subjects and patients with points in test set highlighted.
